# Triaqua-1κ*O*,2κ^2^ *O*-bis­(2,2′-bipyridine)-1κ^2^ *N*,*N*′;2κ^2^ *N*,*N*′-chlorido-1κ*Cl*-μ-terephthalato-1:2κ^2^ *O* ^1^:*O* ^4^-dicopper(II) nitrate monohydrate

**DOI:** 10.1107/S1600536812019848

**Published:** 2012-05-12

**Authors:** Yang Liu, Yong-Lan Feng, Dai-Zhi Kuang

**Affiliations:** a Key Laboratory of Functional Organometallic Materials, Department of Chemistry and Materials Science, Hengyang Normal University, Hengyang 421008, People’s Republic of China

## Abstract

In the binuclear title compound, [Cu_2_(C_8_H_4_O_4_)Cl(C_10_H_8_N_2_)_2_(H_2_O)_3_]NO_3_·H_2_O, the two crystallographically independent Cu^II^ ions have similar coordination environments. One of the Cu^II^ ions has a square-pyramidal arrangement, which is defined by a water mol­ecule occupying the apical position, with the equatorial ligators consisting of two N atoms from a 2,2′-bipyridine mol­ecule, one carboxyl­ate O atom from a terephthalate ligand and one O atom from a water mol­ecule. The other Cu^II^ ion has a similar coordination environment, except that the apical position is occupied by a chloride ligand instead of a water mol­ecule. An O—H⋯O and O—H⋯Cl hydrogen-bonded three-dimensional network is formed between the components.

## Related literature

For related structures, see: Lo *et al.* (2000 ▶); Xu *et al.* (2010 ▶). For background on the use of terephthalic acid and bipyridine as ligands in metal–organic frameworks, see, respectively: Wang *et al.* (2010 ▶); Zhang *et al.* (2010 ▶). 
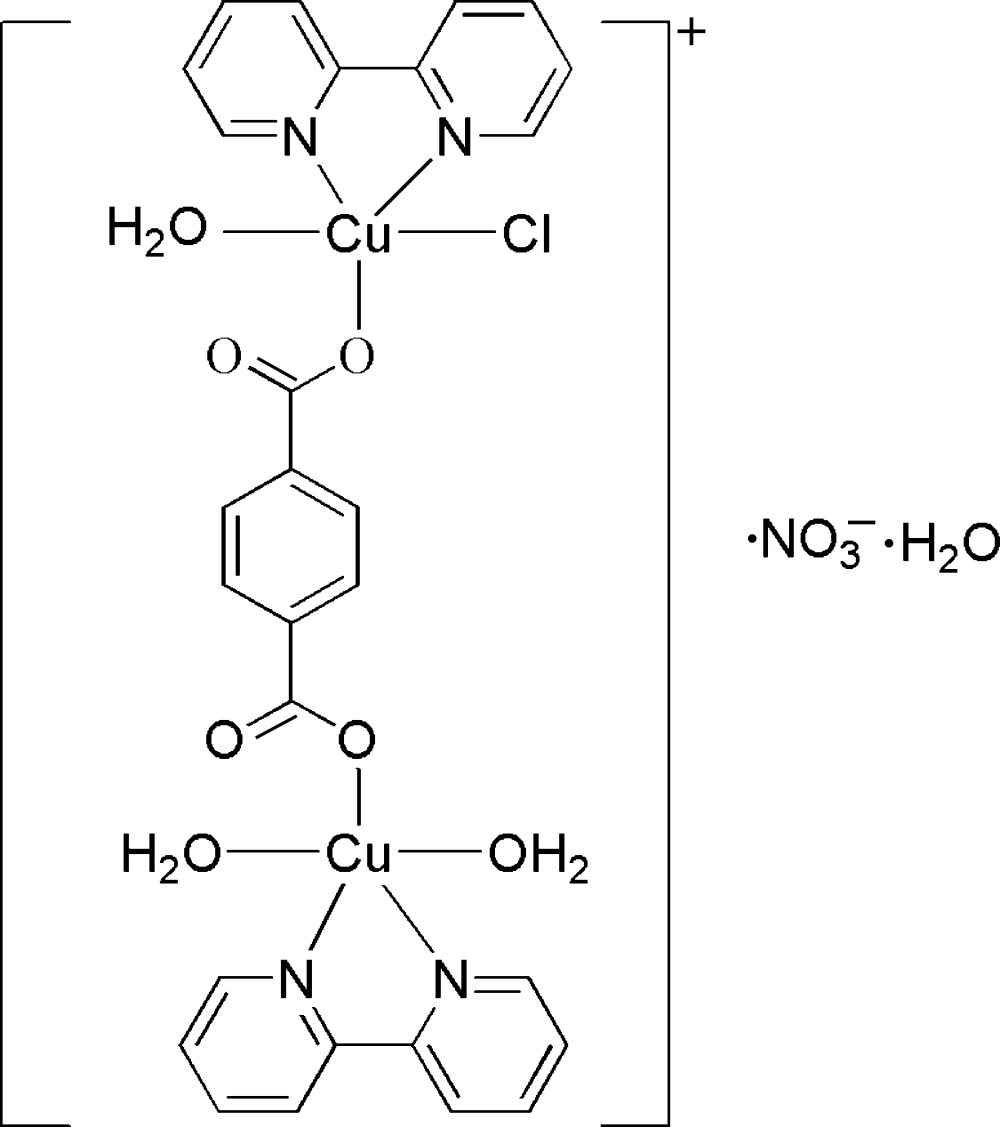


## Experimental

### 

#### Crystal data

[Cu_2_(C_8_H_4_O_4_)Cl(C_10_H_8_N_2_)_2_(H_2_O)_3_]NO_3_·H_2_O
*M* *_r_* = 773.08
Triclinic, 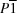

*a* = 10.155 (2) Å
*b* = 11.204 (2) Å
*c* = 15.454 (3) Å
α = 76.34 (3)°
β = 83.61 (3)°
γ = 64.90 (3)°
*V* = 1547.1 (5) Å^3^
*Z* = 2
Mo *K*α radiation
μ = 1.53 mm^−1^
*T* = 293 K
0.20 × 0.20 × 0.20 mm

#### Data collection

Rigaku R-AXIS RAPID IP area-detector diffractometer
Absorption correction: multi-scan (*RAPID-AUTO*; Rigaku, 1998 ▶)) *T*_min_ = 0.750, *T*_max_ = 0.750
15250 measured reflections
6973 independent reflections
4651 reflections with *I* > 2σ(*I*)
*R*_int_ = 0.041

#### Refinement

*R*[*F* ^2^ > 2σ(*F* ^2^)] = 0.052
*wR*(*F* ^2^) = 0.172
*S* = 1.04
6973 reflections
412 parameters
H-atom parameters constrained
Δρ_max_ = 0.71 e Å^−3^
Δρ_min_ = −0.64 e Å^−3^

### 

Data collection: *RAPID-AUTO* (Rigaku, 1998 ▶); cell refinement: *RAPID-AUTO*; data reduction: *CrystalStructure* (Rigaku/MSC, 2004 ▶); program(s) used to solve structure: *SHELXS97* (Sheldrick, 2008 ▶); program(s) used to refine structure: *SHELXL97* (Sheldrick, 2008 ▶); molecular graphics: *SHELXTL* (Sheldrick, 2008 ▶); software used to prepare material for publication: *SHELXL97*.

## Supplementary Material

Crystal structure: contains datablock(s) I, global. DOI: 10.1107/S1600536812019848/fj2542sup1.cif

Structure factors: contains datablock(s) I. DOI: 10.1107/S1600536812019848/fj2542Isup2.hkl

Additional supplementary materials:  crystallographic information; 3D view; checkCIF report

## Figures and Tables

**Table 1:** Hydrogen-bond geometry (Å, °)

*D*—H⋯*A*	*D*—H	H⋯*A*	*D*⋯*A*	*D*—H⋯*A*
O5—H5*A*⋯O4	0.85	2.00	2.534 (4)	120
O6—H6*A*⋯O2	0.86	1.91	2.511 (4)	126
O6—H6*B*⋯O8^i^	0.86	2.34	3.079 (4)	145
O6—H6*B*⋯O10^i^	0.86	2.14	2.923 (4)	151
O7—H7*A*⋯Cl1^ii^	0.85	2.53	3.325 (2)	156
O7—H7*B*⋯O10^iii^	0.86	2.06	2.869 (3)	157
O11—H11*A*⋯O8	0.86	2.26	3.119 (3)	176
O11—H11*A*⋯O9	0.86	2.42	3.038 (3)	129
O11—H11*B*⋯Cl1^iv^	0.84	2.39	3.227 (2)	173

Symmetry codes: (i) 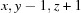; (ii) 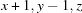; (iii) 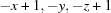; (iv) 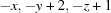.

**Fig. 1.**
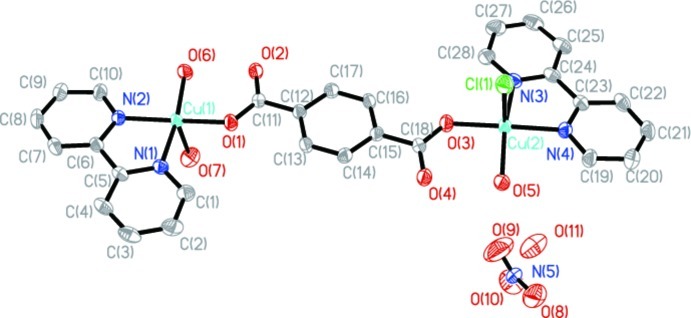
Coordination enviroment of Cu in the complex with hydrogen atoms remove d for the clarity. Displacement ellipsoids are drawn at the 30% probability level.

## supplementary crystallographic information

### Comment

The assembly of mixed-ligand coordination polymers attracted intense attention not only because of their intriguingly complicated architecture, but also potential applications in adsorption, separation(Lo *et al.* 2000), magnetism(Xu *et al.* 2010), and so on. A feasible strategy is to resort to the synergetic coordination of the O-containing and the N-containing ligands. As a rigid and polydentate carboxylato ligand, terephthic acid is extensively used to assembly porous metal-organic-frameworks (Wang *et al.* 2010), while bipyridine ligand is a admirable ancillary ligand and has a conjugate system (Zhang *et al.* 2010). Herein, we synthesized a novel copper complex constructed by terephthalic acid in combination with 2,2'-bipyridine as ancillary ligand.

X-ray diffraction analysis reveals that the title complex crystallizes in the Triclinic group *P-1*. In a assymmetric unit, there are two crystallographically independent copper(II) ions, two terephthalic, two 2,2'-bipyridine moleculars, one nitrate anion, three coordination water and one lattice water molecular. The Cu1(II) ion is a five-coordinated square-pyramidal arrangement which is defined by one coordi nated water molecular occupying the apical position, while the equatorial plane are furnished by two nitrogen atoms from a 2,2'-bipyridine molecular, one carboxylate oxygen atom from terephthlic acid and one oxygen atom from a water molecular. The Cu2(II) ion has a similar coordination enviroment with the Cu1(II)ion except for the apical position occupied by one chlorine anion.Two Cu ions link up by one terephthalic ligand. Interestingly, the benzene ring of the terephthalic ligand has a strong π-π interations with the pyridine ring of 2,2'-bipyridine ligand in the adjacent molecular, which has a 3.355 Å distances between the paralleled faces.

### Experimental

All chemicals were purchased and used without further purification. A mixture of terephtatic acid (0.5 mmol), 2,2'-bipyridine (0.5 mmol) and CuCl_2_2H_2_O (1 mmol) was dissolved in 7 ml of distilled water and a drop of HNO_3_ was add under stirring. Then the resulting mixture was transfered in a 25 ml of Teflon-lined stainless autoclave at 180 °C for 5 days and then cooled to room temperature. Light-blue block crystals of the title compound was obtained.

### Refinement

Accurate unit-cell parameters were determined by a least-squares fit of 2θ values, and intensity data were measured on a rigaku r-axis rapid IP area detector with Mo Kα radiation (λ = 0.71073 Å) at room temperature. The intensities were corrected for Lorentz and polarization effects as well as for empirical absorption based on multi-scan technique; all structures were solved by direct methods and refined by full-matrix least-squares fitting on F^2^ by *SHELX97*(Sheldrick, 2008). All non-hydrogen atoms were refined with anisotropic thermal parameters(Sheldrick, 2008).

### Crystal data

**Table:**

[Cu_2_(C_8_H_4_O_4_)Cl(C_10_H_8_N_2_)_2_(H_2_O)_3_]NO_3_·H_2_O	*Z* = 2
*M**_r_* = 773.08	*F*(000) = 788
Triclinic, *P*1	*D*_x_ = 1.660 Mg m^−^^3^
Hall symbol: -P 1	Mo *K*α radiation, λ = 0.71073 Å
*a* = 10.155 (2) Å	Cell parameters from 9787 reflections
*b* = 11.204 (2) Å	θ = 3.0–27.5°
*c* = 15.454 (3) Å	µ = 1.53 mm^−^^1^
α = 76.34 (3)°	*T* = 293 K
β = 83.61 (3)°	Block, blue
γ = 64.90 (3)°	0.20 × 0.20 × 0.20 mm
*V* = 1547.1 (5) Å^3^	

### Data collection

**Table:**

Rigaku R-AXIS RAPID IP area-detector diffractometer	6973 independent reflections
Radiation source: fine-focus sealed tube	4651 reflections with *I* > 2σ(*I*)
Graphite monochromator	*R*_int_ = 0.041
ω scans	θ_max_ = 27.5°, θ_min_ = 3.2°
Absorption correction: multi-scan (Allen *et al.*, 1991)	*h* = −12→13
*T*_min_ = 0.750, *T*_max_ = 0.750	*k* = −14→14
15250 measured reflections	*l* = −20→20

### Refinement

**Table:**

Refinement on *F*^2^	Primary atom site location: structure-invariant direct methods
Least-squares matrix: full	Secondary atom site location: difference Fourier map
*R*[*F*^2^ > 2σ(*F*^2^)] = 0.052	Hydrogen site location: inferred from neighbouring sites
*wR*(*F*^2^) = 0.172	H-atom parameters constrained
*S* = 1.04	*w* = 1/[σ^2^(*F*_o_^2^) + (0.0996*P*)^2^] where *P* = (*F*_o_^2^ + 2*F*_c_^2^)/3
6973 reflections	(Δ/σ)_max_ = 0.001
412 parameters	Δρ_max_ = 0.71 e Å^−^^3^
0 restraints	Δρ_min_ = −0.64 e Å^−^^3^

### Special details

**Table:**

Geometry. All e.s.d.'s (except the e.s.d. in the dihedral angle between two l.s. planes) are estimated using the full covariance matrix. The cell e.s.d.'s are taken into account individually in the estimation of e.s.d.'s in distances, angles and torsion angles; correlations between e.s.d.'s in cell parameters are only used when they are defined by crystal symmetry. An approximate (isotropic) treatment of cell e.s.d.'s is used for estimating e.s.d.'s involving l.s. planes.
Refinement. Refinement of *F*^2^ against ALL reflections. The weighted *R*-factor *wR* and goodness of fit *S* are based on *F*^2^, conventional *R*-factors *R* are based on *F*, with *F* set to zero for negative *F*^2^. The threshold expression of *F*^2^ > σ(*F*^2^) is used only for calculating *R*-factors(gt) *etc*. and is not relevant to the choice of reflections for refinement. *R*-factors based on *F*^2^ are statistically about twice as large as those based on *F*, and *R*- factors based on ALL data will be even larger.

### Fractional atomic coordinates and isotropic or equivalent isotropic displacement parameters (Å^2^)

**Table:**

	*x*	*y*	*z*	*U*_iso_*/*U*_eq_	
C1	0.7820 (5)	−0.0785 (4)	0.9109 (3)	0.0584 (11)	
H1	0.7098	−0.0158	0.8713	0.070*	
C2	0.9029 (5)	−0.0580 (5)	0.9167 (3)	0.0695 (13)	
H2	0.9134	0.0171	0.8812	0.083*	
C3	1.0111 (5)	−0.1505 (5)	0.9762 (4)	0.0672 (13)	
H3	1.0948	−0.1383	0.9812	0.081*	
C4	0.9918 (4)	−0.2604 (5)	1.0278 (3)	0.0582 (11)	
H4	1.0615	−0.3226	1.0690	0.070*	
C5	0.8687 (4)	−0.2772 (4)	1.0176 (2)	0.0445 (9)	
C6	0.8399 (4)	−0.3956 (4)	1.0644 (2)	0.0433 (9)	
C7	0.9351 (4)	−0.5015 (4)	1.1251 (3)	0.0529 (10)	
H7	1.0217	−0.5010	1.1389	0.063*	
C8	0.8985 (5)	−0.6078 (4)	1.1646 (3)	0.0604 (12)	
H8	0.9589	−0.6789	1.2068	0.072*	
C9	0.7713 (5)	−0.6070 (5)	1.1406 (3)	0.0622 (12)	
H9	0.7459	−0.6784	1.1660	0.075*	
C10	0.6826 (5)	−0.5008 (4)	1.0793 (3)	0.0567 (11)	
H10	0.5975	−0.5014	1.0631	0.068*	
C11	0.3524 (4)	−0.0015 (4)	0.8602 (2)	0.0437 (9)	
C12	0.2979 (4)	0.1371 (4)	0.8007 (2)	0.0409 (8)	
C13	0.3831 (4)	0.2104 (4)	0.7815 (2)	0.0440 (9)	
H13	0.4748	0.1742	0.8063	0.053*	
C14	0.3321 (4)	0.3363 (4)	0.7260 (2)	0.0469 (9)	
H14	0.3890	0.3852	0.7146	0.056*	
C15	0.1970 (4)	0.3906 (4)	0.6871 (2)	0.0401 (8)	
C16	0.1122 (4)	0.3172 (4)	0.7056 (2)	0.0447 (9)	
H16	0.0212	0.3530	0.6797	0.054*	
C17	0.1612 (4)	0.1924 (4)	0.7615 (2)	0.0460 (9)	
H17	0.1033	0.1445	0.7735	0.055*	
C18	0.1432 (4)	0.5279 (4)	0.6280 (2)	0.0439 (9)	
C19	−0.1753 (5)	1.0261 (4)	0.4078 (3)	0.0572 (11)	
H19	−0.0873	1.0215	0.4236	0.069*	
C20	−0.2627 (5)	1.1375 (5)	0.3472 (3)	0.0660 (12)	
H20	−0.2348	1.2078	0.3237	0.079*	
C21	−0.3912 (5)	1.1430 (5)	0.3221 (3)	0.0628 (12)	
H21	−0.4497	1.2157	0.2800	0.075*	
C22	−0.4321 (4)	1.0399 (4)	0.3602 (3)	0.0562 (11)	
H22	−0.5196	1.0425	0.3452	0.067*	
C23	−0.3402 (4)	0.9309 (4)	0.4219 (2)	0.0444 (9)	
C24	−0.3718 (4)	0.8138 (4)	0.4664 (3)	0.0471 (9)	
C25	−0.4963 (4)	0.7998 (5)	0.4544 (3)	0.0578 (11)	
H25	−0.5672	0.8660	0.4154	0.069*	
C26	−0.5148 (5)	0.6861 (5)	0.5011 (4)	0.0688 (13)	
H26	−0.5976	0.6741	0.4933	0.083*	
C27	−0.4091 (5)	0.5916 (5)	0.5591 (3)	0.0707 (14)	
H27	−0.4195	0.5146	0.5911	0.085*	
C28	−0.2876 (5)	0.6112 (4)	0.5696 (3)	0.0612 (11)	
H28	−0.2173	0.5473	0.6099	0.073*	
Cl1	−0.17909 (11)	0.86425 (11)	0.67434 (7)	0.0570 (3)	
Cu1	0.59529 (5)	−0.23100 (4)	0.95432 (3)	0.04425 (17)	
Cu2	−0.09653 (5)	0.75958 (5)	0.53220 (3)	0.04651 (17)	
N1	0.7627 (3)	−0.1860 (3)	0.9601 (2)	0.0457 (7)	
N2	0.7162 (3)	−0.3949 (3)	1.0418 (2)	0.0450 (7)	
N3	−0.2673 (3)	0.7199 (3)	0.5236 (2)	0.0462 (7)	
N4	−0.2143 (3)	0.9249 (3)	0.4442 (2)	0.0473 (8)	
N5	0.3000 (4)	0.6385 (4)	0.1813 (2)	0.0597 (10)	
O1	0.4807 (3)	−0.0500 (3)	0.88775 (19)	0.0536 (7)	
O2	0.2685 (3)	−0.0594 (3)	0.8763 (2)	0.0637 (9)	
O3	0.0152 (3)	0.5775 (3)	0.59884 (19)	0.0547 (7)	
O4	0.2273 (3)	0.5869 (3)	0.6131 (2)	0.0639 (9)	
O5	0.0782 (3)	0.7958 (3)	0.5013 (2)	0.0637 (9)	
O6	0.4297 (3)	−0.2800 (3)	0.9705 (2)	0.0651 (9)	
O7	0.6810 (2)	−0.31174 (19)	0.82969 (13)	0.0757 (9)	
O8	0.3190 (2)	0.73970 (19)	0.16260 (13)	0.1204 (16)	
O9	0.2562 (2)	0.60351 (19)	0.25133 (13)	0.183 (3)	
O10	0.3218 (2)	0.57418 (19)	0.12290 (13)	0.1219 (16)	
O11	0.1857 (2)	0.84194 (19)	0.33688 (13)	0.1176 (16)	
H5A	0.1636	0.7593	0.5220	0.141*	
H5B	0.0846	0.7747	0.4504	0.141*	
H6A	0.3714	−0.2463	0.9265	0.141*	
H6B	0.3798	−0.2936	1.0171	0.141*	
H7A	0.7169	−0.2841	0.7805	0.141*	
H7B	0.6878	−0.3903	0.8283	0.141*	
H11A	0.2265	0.8103	0.2905	0.141*	
H11B	0.1831	0.9200	0.3289	0.141*	

### Atomic displacement parameters (Å^2^)

**Table:**

	*U*^11^	*U*^22^	*U*^33^	*U*^12^	*U*^13^	*U*^23^
C1	0.054 (2)	0.057 (3)	0.072 (3)	−0.033 (2)	−0.004 (2)	−0.006 (2)
C2	0.069 (3)	0.069 (3)	0.090 (3)	−0.047 (3)	0.006 (3)	−0.020 (3)
C3	0.045 (2)	0.076 (3)	0.100 (4)	−0.035 (2)	0.006 (2)	−0.038 (3)
C4	0.038 (2)	0.076 (3)	0.068 (3)	−0.022 (2)	−0.0039 (19)	−0.031 (2)
C5	0.0328 (19)	0.053 (2)	0.050 (2)	−0.0153 (16)	0.0002 (15)	−0.0207 (18)
C6	0.0358 (19)	0.052 (2)	0.0386 (18)	−0.0123 (16)	−0.0024 (15)	−0.0136 (17)
C7	0.040 (2)	0.057 (2)	0.051 (2)	−0.0039 (18)	−0.0099 (17)	−0.018 (2)
C8	0.062 (3)	0.052 (2)	0.044 (2)	−0.002 (2)	−0.0144 (19)	−0.003 (2)
C9	0.069 (3)	0.056 (3)	0.055 (2)	−0.025 (2)	−0.007 (2)	0.004 (2)
C10	0.057 (3)	0.049 (2)	0.059 (2)	−0.025 (2)	−0.012 (2)	0.008 (2)
C11	0.040 (2)	0.0400 (19)	0.0444 (19)	−0.0133 (16)	−0.0021 (15)	−0.0018 (17)
C12	0.040 (2)	0.0375 (18)	0.0428 (19)	−0.0143 (15)	−0.0020 (15)	−0.0071 (16)
C13	0.0378 (19)	0.0365 (19)	0.052 (2)	−0.0103 (15)	−0.0084 (16)	−0.0046 (17)
C14	0.047 (2)	0.046 (2)	0.051 (2)	−0.0242 (17)	−0.0042 (17)	−0.0021 (18)
C15	0.0408 (19)	0.0366 (18)	0.0383 (18)	−0.0124 (15)	−0.0001 (15)	−0.0063 (16)
C16	0.0362 (19)	0.040 (2)	0.051 (2)	−0.0108 (15)	−0.0090 (16)	−0.0032 (17)
C17	0.045 (2)	0.044 (2)	0.051 (2)	−0.0223 (17)	−0.0038 (17)	−0.0056 (18)
C18	0.038 (2)	0.040 (2)	0.048 (2)	−0.0117 (16)	−0.0022 (16)	−0.0053 (17)
C19	0.054 (2)	0.055 (2)	0.058 (2)	−0.025 (2)	−0.0182 (19)	0.009 (2)
C20	0.075 (3)	0.056 (3)	0.057 (3)	−0.023 (2)	−0.012 (2)	0.003 (2)
C21	0.059 (3)	0.058 (3)	0.050 (2)	−0.007 (2)	−0.017 (2)	0.002 (2)
C22	0.043 (2)	0.064 (3)	0.052 (2)	−0.0077 (19)	−0.0133 (18)	−0.016 (2)
C23	0.038 (2)	0.053 (2)	0.0393 (18)	−0.0120 (17)	−0.0017 (15)	−0.0176 (17)
C24	0.039 (2)	0.056 (2)	0.049 (2)	−0.0161 (17)	0.0027 (16)	−0.0249 (19)
C25	0.038 (2)	0.076 (3)	0.066 (3)	−0.020 (2)	−0.0041 (19)	−0.030 (2)
C26	0.044 (3)	0.081 (3)	0.096 (4)	−0.034 (2)	0.005 (2)	−0.033 (3)
C27	0.069 (3)	0.067 (3)	0.094 (4)	−0.046 (3)	0.015 (3)	−0.022 (3)
C28	0.055 (3)	0.053 (2)	0.077 (3)	−0.027 (2)	0.002 (2)	−0.008 (2)
Cl1	0.0546 (6)	0.0614 (6)	0.0501 (5)	−0.0198 (5)	−0.0087 (4)	−0.0068 (5)
Cu1	0.0352 (3)	0.0418 (3)	0.0544 (3)	−0.0203 (2)	−0.0121 (2)	0.0060 (2)
Cu2	0.0362 (3)	0.0433 (3)	0.0569 (3)	−0.0192 (2)	−0.0132 (2)	0.0064 (2)
N1	0.0398 (17)	0.0490 (18)	0.0527 (18)	−0.0235 (14)	−0.0049 (14)	−0.0068 (15)
N2	0.0414 (17)	0.0428 (17)	0.0480 (17)	−0.0185 (14)	−0.0081 (13)	0.0015 (15)
N3	0.0364 (16)	0.0481 (18)	0.0557 (18)	−0.0184 (14)	−0.0024 (14)	−0.0110 (16)
N4	0.0433 (18)	0.0473 (18)	0.0467 (17)	−0.0185 (14)	−0.0095 (14)	0.0022 (15)
N5	0.063 (2)	0.054 (2)	0.061 (2)	−0.0309 (19)	0.0025 (18)	0.0004 (19)
O1	0.0440 (15)	0.0427 (15)	0.0704 (18)	−0.0214 (12)	−0.0156 (13)	0.0087 (14)
O2	0.0460 (16)	0.0582 (18)	0.080 (2)	−0.0285 (14)	−0.0166 (14)	0.0198 (16)
O3	0.0453 (15)	0.0418 (14)	0.0716 (18)	−0.0203 (12)	−0.0163 (13)	0.0097 (14)
O4	0.0499 (17)	0.0563 (17)	0.080 (2)	−0.0284 (14)	−0.0187 (15)	0.0174 (16)
O5	0.0411 (15)	0.0654 (19)	0.078 (2)	−0.0273 (14)	−0.0164 (14)	0.0138 (16)
O6	0.0430 (15)	0.0609 (18)	0.089 (2)	−0.0323 (14)	−0.0151 (14)	0.0168 (17)
O7	0.096 (3)	0.067 (2)	0.073 (2)	−0.0429 (19)	−0.0019 (18)	−0.0145 (17)
O8	0.142 (4)	0.080 (3)	0.156 (4)	−0.064 (3)	0.007 (3)	−0.027 (3)
O9	0.225 (7)	0.198 (6)	0.123 (4)	−0.118 (6)	0.055 (4)	0.004 (4)
O10	0.114 (4)	0.109 (4)	0.155 (5)	−0.040 (3)	−0.007 (3)	−0.060 (3)
O11	0.174 (5)	0.100 (3)	0.095 (3)	−0.079 (3)	0.039 (3)	−0.027 (3)

### Geometric parameters (Å, º)

**Table:**

C1—N1	1.342 (5)	C20—C21	1.374 (6)
C1—C2	1.358 (6)	C20—H20	0.9300
C1—H1	0.9300	C21—C22	1.373 (6)
C2—C3	1.391 (7)	C21—H21	0.9300
C2—H2	0.9300	C22—C23	1.397 (5)
C3—C4	1.377 (6)	C22—H22	0.9300
C3—H3	0.9300	C23—N4	1.330 (5)
C4—C5	1.371 (5)	C23—C24	1.476 (5)
C4—H4	0.9300	C24—N3	1.355 (5)
C5—N1	1.358 (5)	C24—C25	1.375 (6)
C5—C6	1.481 (5)	C25—C26	1.385 (6)
C6—N2	1.338 (5)	C25—H25	0.9300
C6—C7	1.388 (5)	C26—C27	1.370 (7)
C7—C8	1.382 (6)	C26—H26	0.9300
C7—H7	0.9300	C27—C28	1.374 (6)
C8—C9	1.379 (6)	C27—H27	0.9300
C8—H8	0.9300	C28—N3	1.342 (5)
C9—C10	1.368 (6)	C28—H28	0.9300
C9—H9	0.9300	Cl1—Cu2	2.6222 (14)
C10—N2	1.355 (5)	Cu1—O1	1.950 (3)
C10—H10	0.9300	Cu1—O6	1.954 (3)
C11—O2	1.246 (4)	Cu1—N1	1.983 (3)
C11—O1	1.260 (4)	Cu1—N2	1.997 (3)
C11—C12	1.507 (5)	Cu1—O7	2.251 (2)
C12—C13	1.393 (5)	Cu2—O3	1.953 (3)
C12—C17	1.400 (5)	Cu2—O5	1.967 (3)
C13—C14	1.379 (5)	Cu2—N3	1.989 (3)
C13—H13	0.9300	Cu2—N4	2.001 (3)
C14—C15	1.385 (5)	N5—O9	1.167 (4)
C14—H14	0.9300	N5—O8	1.194 (4)
C15—C16	1.392 (5)	N5—O10	1.230 (5)
C15—C18	1.495 (5)	O5—H5A	0.8491
C16—C17	1.372 (5)	O5—H5B	0.8602
C16—H16	0.9300	O6—H6A	0.8570
C17—H17	0.9300	O6—H6B	0.8543
C18—O4	1.257 (4)	O7—H7A	0.8545
C18—O3	1.265 (4)	O7—H7B	0.8581
C19—N4	1.337 (5)	O11—H11A	0.8600
C19—C20	1.384 (6)	O11—H11B	0.8431
C19—H19	0.9300		
			
N1—C1—C2	122.5 (4)	N4—C23—C22	121.5 (4)
N1—C1—H1	118.8	N4—C23—C24	114.7 (3)
C2—C1—H1	118.8	C22—C23—C24	123.8 (4)
C1—C2—C3	119.3 (4)	N3—C24—C25	121.7 (4)
C1—C2—H2	120.3	N3—C24—C23	113.7 (3)
C3—C2—H2	120.3	C25—C24—C23	124.5 (4)
C4—C3—C2	118.7 (4)	C24—C25—C26	119.2 (4)
C4—C3—H3	120.6	C24—C25—H25	120.4
C2—C3—H3	120.6	C26—C25—H25	120.4
C5—C4—C3	119.3 (4)	C27—C26—C25	118.8 (4)
C5—C4—H4	120.4	C27—C26—H26	120.6
C3—C4—H4	120.4	C25—C26—H26	120.6
N1—C5—C4	121.8 (4)	C26—C27—C28	119.7 (4)
N1—C5—C6	113.7 (3)	C26—C27—H27	120.2
C4—C5—C6	124.5 (4)	C28—C27—H27	120.2
N2—C6—C7	122.0 (4)	N3—C28—C27	122.0 (4)
N2—C6—C5	114.8 (3)	N3—C28—H28	119.0
C7—C6—C5	123.2 (4)	C27—C28—H28	119.0
C8—C7—C6	118.6 (4)	O1—Cu1—O6	92.65 (12)
C8—C7—H7	120.7	O1—Cu1—N1	91.80 (12)
C6—C7—H7	120.7	O6—Cu1—N1	170.29 (13)
C9—C8—C7	119.1 (4)	O1—Cu1—N2	167.42 (13)
C9—C8—H8	120.5	O6—Cu1—N2	92.88 (12)
C7—C8—H8	120.5	N1—Cu1—N2	81.12 (13)
C10—C9—C8	119.8 (4)	O1—Cu1—O7	92.92 (11)
C10—C9—H9	120.1	O6—Cu1—O7	95.55 (12)
C8—C9—H9	120.1	N1—Cu1—O7	92.84 (11)
N2—C10—C9	121.5 (4)	N2—Cu1—O7	97.78 (11)
N2—C10—H10	119.3	O3—Cu2—O5	92.61 (12)
C9—C10—H10	119.3	O3—Cu2—N3	92.51 (12)
O2—C11—O1	125.6 (3)	O5—Cu2—N3	162.04 (14)
O2—C11—C12	117.6 (3)	O3—Cu2—N4	167.36 (14)
O1—C11—C12	116.8 (3)	O5—Cu2—N4	91.12 (12)
C13—C12—C17	118.9 (3)	N3—Cu2—N4	80.46 (13)
C13—C12—C11	121.3 (3)	O3—Cu2—Cl1	94.28 (10)
C17—C12—C11	119.8 (3)	O5—Cu2—Cl1	98.55 (11)
C14—C13—C12	120.4 (3)	N3—Cu2—Cl1	98.21 (10)
C14—C13—H13	119.8	N4—Cu2—Cl1	97.10 (11)
C12—C13—H13	119.8	C1—N1—C5	118.4 (3)
C13—C14—C15	120.6 (3)	C1—N1—Cu1	126.3 (3)
C13—C14—H14	119.7	C5—N1—Cu1	115.3 (3)
C15—C14—H14	119.7	C6—N2—C10	119.0 (3)
C14—C15—C16	119.1 (3)	C6—N2—Cu1	115.0 (2)
C14—C15—C18	119.7 (3)	C10—N2—Cu1	126.0 (3)
C16—C15—C18	121.2 (3)	C28—N3—C24	118.5 (4)
C17—C16—C15	120.7 (3)	C28—N3—Cu2	126.0 (3)
C17—C16—H16	119.6	C24—N3—Cu2	115.6 (3)
C15—C16—H16	119.6	C23—N4—C19	119.5 (3)
C16—C17—C12	120.3 (3)	C23—N4—Cu2	115.6 (3)
C16—C17—H17	119.9	C19—N4—Cu2	124.9 (3)
C12—C17—H17	119.9	O9—N5—O8	121.9 (4)
O4—C18—O3	125.0 (3)	O9—N5—O10	120.2 (3)
O4—C18—C15	117.4 (3)	O8—N5—O10	117.8 (3)
O3—C18—C15	117.6 (3)	C11—O1—Cu1	128.7 (2)
N4—C19—C20	121.6 (4)	C18—O3—Cu2	128.4 (2)
N4—C19—H19	119.2	Cu2—O5—H5A	133.7
C20—C19—H19	119.2	Cu2—O5—H5B	90.8
C21—C20—C19	119.3 (4)	H5A—O5—H5B	106.8
C21—C20—H20	120.4	Cu1—O6—H6A	115.0
C19—C20—H20	120.4	Cu1—O6—H6B	130.8
C22—C21—C20	119.1 (4)	H6A—O6—H6B	106.7
C22—C21—H21	120.5	Cu1—O7—H7A	134.4
C20—C21—H21	120.5	Cu1—O7—H7B	118.9
C21—C22—C23	119.0 (4)	H7A—O7—H7B	106.5
C21—C22—H22	120.5	H11A—O11—H11B	107.4
C23—C22—H22	120.5		
			
N1—C1—C2—C3	−0.6 (8)	N2—Cu1—N1—C5	−1.9 (3)
C1—C2—C3—C4	−0.1 (8)	O7—Cu1—N1—C5	−99.3 (3)
C2—C3—C4—C5	1.4 (7)	C7—C6—N2—C10	0.1 (6)
C3—C4—C5—N1	−2.2 (6)	C5—C6—N2—C10	178.2 (4)
C3—C4—C5—C6	175.6 (4)	C7—C6—N2—Cu1	179.3 (3)
N1—C5—C6—N2	1.0 (5)	C5—C6—N2—Cu1	−2.5 (4)
C4—C5—C6—N2	−177.0 (4)	C9—C10—N2—C6	1.0 (7)
N1—C5—C6—C7	179.1 (4)	C9—C10—N2—Cu1	−178.2 (4)
C4—C5—C6—C7	1.1 (6)	O1—Cu1—N2—C6	−53.9 (7)
N2—C6—C7—C8	−1.5 (6)	O6—Cu1—N2—C6	−169.9 (3)
C5—C6—C7—C8	−179.5 (4)	N1—Cu1—N2—C6	2.4 (3)
C6—C7—C8—C9	1.9 (7)	O7—Cu1—N2—C6	94.1 (3)
C7—C8—C9—C10	−0.9 (7)	O1—Cu1—N2—C10	125.3 (5)
C8—C9—C10—N2	−0.6 (7)	O6—Cu1—N2—C10	9.3 (4)
O2—C11—C12—C13	178.0 (4)	N1—Cu1—N2—C10	−178.4 (4)
O1—C11—C12—C13	−3.5 (6)	O7—Cu1—N2—C10	−86.7 (4)
O2—C11—C12—C17	−3.9 (6)	C27—C28—N3—C24	1.3 (7)
O1—C11—C12—C17	174.6 (4)	C27—C28—N3—Cu2	−179.7 (4)
C17—C12—C13—C14	1.0 (6)	C25—C24—N3—C28	−0.4 (6)
C11—C12—C13—C14	179.1 (4)	C23—C24—N3—C28	177.9 (4)
C12—C13—C14—C15	−1.3 (6)	C25—C24—N3—Cu2	−179.4 (3)
C13—C14—C15—C16	0.8 (6)	C23—C24—N3—Cu2	−1.1 (4)
C13—C14—C15—C18	179.3 (4)	O3—Cu2—N3—C28	12.5 (4)
C14—C15—C16—C17	−0.1 (6)	O5—Cu2—N3—C28	119.0 (5)
C18—C15—C16—C17	−178.5 (4)	N4—Cu2—N3—C28	−178.1 (4)
C15—C16—C17—C12	−0.2 (6)	Cl1—Cu2—N3—C28	−82.2 (4)
C13—C12—C17—C16	−0.3 (6)	O3—Cu2—N3—C24	−168.5 (3)
C11—C12—C17—C16	−178.4 (4)	O5—Cu2—N3—C24	−62.1 (5)
C14—C15—C18—O4	2.8 (6)	N4—Cu2—N3—C24	0.9 (3)
C16—C15—C18—O4	−178.7 (4)	Cl1—Cu2—N3—C24	96.8 (3)
C14—C15—C18—O3	−175.1 (4)	C22—C23—N4—C19	1.0 (6)
C16—C15—C18—O3	3.3 (6)	C24—C23—N4—C19	−179.9 (4)
N4—C19—C20—C21	−1.5 (8)	C22—C23—N4—Cu2	−179.1 (3)
C19—C20—C21—C22	2.2 (7)	C24—C23—N4—Cu2	0.1 (4)
C20—C21—C22—C23	−1.4 (7)	C20—C19—N4—C23	−0.1 (7)
C21—C22—C23—N4	−0.2 (6)	C20—C19—N4—Cu2	180.0 (4)
C21—C22—C23—C24	−179.3 (4)	O3—Cu2—N4—C23	56.4 (7)
N4—C23—C24—N3	0.7 (5)	O5—Cu2—N4—C23	163.5 (3)
C22—C23—C24—N3	179.9 (4)	N3—Cu2—N4—C23	−0.5 (3)
N4—C23—C24—C25	178.9 (4)	Cl1—Cu2—N4—C23	−97.7 (3)
C22—C23—C24—C25	−1.9 (6)	O3—Cu2—N4—C19	−123.7 (6)
N3—C24—C25—C26	−0.8 (6)	O5—Cu2—N4—C19	−16.5 (4)
C23—C24—C25—C26	−178.9 (4)	N3—Cu2—N4—C19	179.4 (4)
C24—C25—C26—C27	1.0 (7)	Cl1—Cu2—N4—C19	82.2 (4)
C25—C26—C27—C28	−0.1 (8)	O2—C11—O1—Cu1	5.6 (7)
C26—C27—C28—N3	−1.1 (8)	C12—C11—O1—Cu1	−172.8 (2)
C2—C1—N1—C5	−0.1 (7)	O6—Cu1—O1—C11	−7.5 (4)
C2—C1—N1—Cu1	−177.4 (4)	N1—Cu1—O1—C11	−178.9 (4)
C4—C5—N1—C1	1.5 (6)	N2—Cu1—O1—C11	−123.5 (5)
C6—C5—N1—C1	−176.5 (4)	O7—Cu1—O1—C11	88.2 (4)
C4—C5—N1—Cu1	179.1 (3)	O4—C18—O3—Cu2	−5.3 (6)
C6—C5—N1—Cu1	1.0 (4)	C15—C18—O3—Cu2	172.4 (3)
O1—Cu1—N1—C1	−15.0 (4)	O5—Cu2—O3—C18	16.7 (4)
N2—Cu1—N1—C1	175.5 (4)	N3—Cu2—O3—C18	179.5 (4)
O7—Cu1—N1—C1	78.1 (4)	N4—Cu2—O3—C18	123.7 (6)
O1—Cu1—N1—C5	167.7 (3)	Cl1—Cu2—O3—C18	−82.1 (4)

### Hydrogen-bond geometry (Å, º)

**Table:**

*D*—H···*A*	*D*—H	H···*A*	*D*···*A*	*D*—H···*A*
O5—H5*A*···O4	0.85	2.00	2.534 (4)	120
O6—H6*A*···O2	0.86	1.91	2.511 (4)	126
O6—H6*B*···O8^i^	0.86	2.34	3.079 (4)	145
O6—H6*B*···O10^i^	0.86	2.14	2.923 (4)	151
O7—H7*A*···Cl1^ii^	0.85	2.53	3.325 (2)	156
O7—H7*B*···O10^iii^	0.86	2.06	2.869 (3)	157
O11—H11*A*···O8	0.86	2.26	3.119 (3)	176
O11—H11*A*···O9	0.86	2.42	3.038 (3)	129
O11—H11*B*···Cl1^iv^	0.84	2.39	3.227 (2)	173

Symmetry codes: (i) *x*, *y*−1, *z*+1; (ii) *x*+1, *y*−1, *z*; (iii) −*x*+1, −*y*, −*z*+1; (iv) −*x*, −*y*+2, −*z*+1.
